# Elevated Levels of Apolipoprotein CIII Increase the Risk of Postprandial Hypertriglyceridemia

**DOI:** 10.3389/fendo.2021.646185

**Published:** 2021-04-23

**Authors:** Yunpeng Guan, Xiaoyu Hou, Peipei Tian, Luping Ren, Yong Tang, An Song, Jiajun Zhao, Ling Gao, Guangyao Song

**Affiliations:** ^1^ Department of Internal Medicine, Hebei Medical University, Shijiazhuang, China; ^2^ Department of Endocrinology, Hebei General Hospital, Shijiazhuang, China; ^3^ Department of Endocrinology, Cangzhou Central Hospital, Cangzhou, China; ^4^ Key Laboratory of Endocrinology, Ministry of Health, Department of Endocrinology, Peking Union Medical College Hospital, Peking Union Medical College, Chinese Academy of Medical Sciences, Beijing, China; ^5^ Department of Endocrinology and Metabolism, Shandong Provincial Hospital Affiliated to Shandong University, Jinan, China

**Keywords:** triglycerides, dyslipidemia, postprandial hypertriglyceridemia, triglyceride-rich lipoprotein remnants, apolipoprotein CIII

## Abstract

**Background:**

To investigate possible mechanisms of postprandial hypertriglyceridemia (PPT), we analyzed serum lipid and apolipoprotein (Apo) AI, B, CII and CIII levels before and after a high-fat meal.

**Methods:**

The study has been registered with the China Clinical Trial Registry (registration number:ChiCTR1800019514; URL: http://www.chictr.org.cn/index.aspx). We recruited 143 volunteers with normal fasting triglyceride (TG) levels. All subjects consumed a high-fat test meal. Venous blood samples were obtained during fasting and at 2, 4, and 6 hours after the high-fat meal. PPT was defined as TG ≥2.5 mmol/L any time after the meal. Subjects were divided into two groups according to the high-fat meal test results: postprandial normal triglyceride (PNT) and PPT. We compared the fasting and postprandial lipid and ApoAI, ApoB, ApoCII and ApoCIII levels between the two groups.

**Results:**

Significant differences were found between the groups in fasting insulin, homeostasis model assessment of insulin resistance (HOMA-IR), TG, total cholesterol (TC), low-density lipoprotein cholesterol (LDL-C), non-high-density lipoprotein cholesterol (non-HDL-C), TG-rich lipoprotein remnants (TRLRs), ApoB, ApoCIII, ApoAI/ApoB and ApoCII/ApoCIII. The insulin, HOMA-IR, TG, TC, LDL-C, non-HDL-C, TRLRs, ApoB, ApoCIII and ApoCII/ApoCIII values were higher in the PPT group, while the ApoAI/ApoB ratio was higher in the PNT group. The postprandial TG level peaked in the PNT group 2 hours after the meal but was significantly higher in the PPT group and peaked at 4 hours. TRLRs gradually increased within 6 hours after the high-fat meal in both groups. The area under the curve (AUC) of TG and TRLRs and the AUC increment were higher in the PPT group (*P *< 0.001). ApoCIII peaked in the PNT group 2 hours after the meal and gradually decreased. ApoCIII gradually increased in the PPT group within 6 hours after the meal, exhibiting a greater AUC increment (*P *< 0.001). Fasting ApoCIII was positively correlated with age, systolic and diastolic blood pressure, body mass index (BMI), waist circumference, TC, TG, LDL-C, non-HDL-C, TRLRs, and ApoB (*P*<0.05). ApoCIII was an independent risk factor of PPT after adjustment for BMI, waist circumference, TC, LDL-C, and ApoB (*P* < 0.001, OR=1.188).

**Conclusions:**

Elevated ApoCIII levels may cause PPT.

## Introduction

Recently, changes in lifestyle and diet structure have led to an increasing incidence of hyperlipidemia. Epidemiological studies have shown that elevated triglyceride (TG) levels increase the risk of cardiovascular and metabolic diseases. Early detection of abnormal TG metabolism is of great significance for the prevention of many diseases. Typically, serum lipid levels are monitored in the fasting state, but many studies have confirmed that postprandial TG levels are closely related to various diseases such as atherosclerosis (1), coronary heart disease (2), and type 2 diabetes (3). Postprandial hypertriglyceridemia (PPT) is a condition in which plasma TG and TG-rich lipoprotein remnants (TRLRs) are increased during the postprandial period. TRLRs mainly include the hydrolysis remnants of chylomicron (CM), very low-density lipoprotein (VLDL), and intermediate-density lipoprotein (IDL). Investigation of the pathogenesis of PPT may provide new insights for prevention and treatment of cardiovascular and metabolic diseases.

Lipids are transported in the form of lipoproteins. Apolipoproteins (Apos), as the proteins in lipoproteins, play an important role in lipid metabolism. ApoAI is present in high-density lipoprotein cholesterol (HDL-C). It can activate lecithin:cholesterol acyl transferase (LCAT), a key enzyme in reverse transport of cholesterol, and maintains the function of HDL-C. Circulating ApoB mainly consists of ApoB100 and B48. ApoB100 is synthesized in the liver, while ApoB48 is produced in the intestine. ApoB48 is required for CM production, and ApoB100 is an essential structural component of VLDL and its metabolic products, IDL and LDL, as well as lipoprotein(a). ApoC is a water-soluble protein with four subtypes: CI, CII, CIII, and CIV. ApoCII is a known activator of lipoprotein lipase (LPL) (4), which promotes the hydrolysis of CM and VLDL. ApoCIII is an important regulator of TG metabolism (5, 6). It inhibits LPL (7) and affects receptor-mediated hepatic uptake of TRLRs (8). At higher concentrations, ApoCIII also affects the activity of hepatic lipase (9), which plays an important role in the conversion of VLDL to IDL and low-density lipoprotein (LDL). Mice overexpressing the human ApoCIII gene were fed with different diets, and the increase in ApoCIII was shown to aggravate diet-related obesity (10). Many causes, such as saturated fatty acid in the diet (11), accumulation of visceral adipose tissue (12) and insulin resistance (13), may increase postprandial TG levels, but research on the influence of Apos on postprandial TG levels is rare. This study aims to investigate whether PPT is related to ApoAI, ApoB, ApoCII, and ApoCIII levels using a high-fat meal test to provide a theoretical basis for intervention in dyslipidemia.

## Materials and Methods

### Participant Recruitment

This study was approved by the Ethics Committee of Hebei General Hospital. From May 2017 to February 2018, volunteers were recruited from the endocrinology clinic. All volunteers signed an informed consent form. The study has been registered with the China Clinical Trial Registry (registration number: ChiCTR1800019514).

The following were exclusion criteria for participants: smokers, drinkers (The alcohol intake was more than 175g for male participants in a week and 105g for females), vegetarian diet, malignant tumors, individuals with the family history of dyslipidemia, digestive system diseases (hepatobiliary pancreatic spleen disease), heart disease, abnormal thyroid function, diabetes, fasting dyslipidemia, kidney disease, acute and chronic blood disease, infectious disease, mental disorders, use of drugs that affect lipid metabolism and inflammation (fish oil, contraceptives, hormones, beta blockers, diuretics), severe infections, stroke, pregnancy, and changes in weight of more than 3 kg in the past 3 months.

### Study Design

Height, weight, waist circumference (WC), and blood pressure were measured in volunteers by professional physicians. WC was measured according to World Health Organization (WHO) criteria (14). The body mass index (BMI) was calculated as the weight in kilograms divided by height in meters squared (kg/m^2^). All volunteers completed detection of blood lipids and an oral glucose tolerance test (OGTT). Participants with type 2 diabetes according to the results of the OGTT were excluded. The diagnosis of type 2 diabetes was determined according to the 1999 WHO criteria. Individuals with normal fasting lipid levels (TG <1.7 mmol/L; total cholesterol (TC) <5.2 mmol/L; low-density lipoprotein cholesterol (LDL-C) <3.4 mmol/L; HDL-C >1 mmol/L) were recruited according to the diagnostic criteria of the Chinese Guidelines for the Prevention and Treatment of Adult Dyslipidemia in 2016 (15).

All of the included participants had consumed an ordinary diet (300 to 500 grams of cereals, 400 grams of vegetables, 100 grams of fruits, 50 to 100 grams of meat, 25 to 50 grams of eggs, 100 grams of milk and 25 grams of fat in a day) within 1 week before the high-fat meal test, and smoking and drinking were prohibited. The participants ate a high-fat meal in the early morning (after fasting for more than 8 hours). The high-fat meal was prepared by professional nutritionists. The high-fat meal contained 1500kcal, including fat (monounsaturated fatty acid:polyunsaturated fatty acid:saturated fatty acid=2:2:1), carbohydrates, and protein, which provided 60%, 20%, and 20% of the total calories. All participants finished eating within 10 minutes and stopped eating any food within 6 hours; participants were permitted to drink freely, but smoking and strenuous exercise were prohibited. The participants’ venous blood was drawn during fasting and at 2, 4, and 6 hours after the high-fat meal. Plasma was separated after whole blood was centrifuged at 3000 rpm for 10 minutes and then stored at −80°C until the time of assay.

Fasting and postprandial blood were drawn for TG, TC, HDL-C, LDL-C, ApoAI, ApoB, ApoCII and ApoCIII measurement. ApoCII and ApoCIII were measured with an enzyme-linked immunosorbent assay. The other indicators were measured with an automatic biochemical analyzer by professional laboratory physicians. Non-HDL-C and TRLRs were estimated by the following formulas, non-HDL-C=TC−(HDL-C) and TRLRs=TC−(HDL-C)−(LDL-C).

The subjects were divided into two groups according to the high-fat meal test results: ① postprandial normal triglyceride (PNT) and ②PPT. With reference to the consensus reached at the conference on the correlation between pre- and postprandial TG levels and cardiovascular disease risk held in 2010 (16) and the expert panel statement in 2019 (17), a TG level ≥2.5 mmol/L at any time after the high-fat meal was used as the criterion for PPT.

### Statistical Analysis

SPSS25.0 software (SPSS Inc, IBM Corporation, Armonk, New York, USA) was used for the statistical analysis. Normally distributed data are expressed as the mean ± standard deviation (SD), and non-normally distributed data are represented by the median (interquartile range). For comparisons between two groups, if the data conformed to a normal distribution and the variances were uniform, an unpaired t-test was used; otherwise, a Mann-Whitney test was used. Categorical variables were compared using chi-squared statistical tests. The area under the curve (AUC) and the change in the AUC (ΔAUC), representing an increase in area after the high-fat meal compared with the fasting levels, were estimated by the trapezoid method. Pearson correlation was performed to determine the correlation strength between variables that were normally distributed; otherwise, Spearman correlation was used. Possible risk factors of PPT were analyzed using univariable logistic regression followed by multivariable logistic regression. All results are based on a value of *P *< 0.05 as the standard of statistical significance.

## Results

### Anthropometric and Fasting Metabolic Parameters in the Two Groups

A total of 143 subjects were recruited in this study, including 56 in the PNT group and 87 in the PPT group. There was no significant difference in age or gender between the two groups (**Table 1**). The WC, BMI and systolic blood pressure were higher in the PPT group than those in the PNT group. Significant differences were observed in fasting insulin, homeostasis model assessment of insulin resistance (HOMA-IR), TG, TC, LDL-C, non-HDL-C, TRLRs, ApoB, ApoCIII, ApoAI/ApoB and ApoCII/ApoCIII values between the two groups. The insulin, HOMA-IR, TG, TC, LDL-C, non-HDL-C, TRLRs, ApoB, and ApoCIII levels and the ApoCII/ApoCIII ratio were higher in the PPT group than those in the PNT group, while the ApoAI/ApoB ratio was higher in the PNT group. There was no significant difference in fasting glucose, HDL-C, ApoAI, and ApoCII levels between the two groups.

**Table 1:** Anthropometric and fasting metabolic parameters in the two groups.

	PNT (56)	PPT (87)	*P*
Age (years)	44.18 ± 5.94	45.11 ± 5.26	0.325
Male/Female	29/27	45/42	0.994
WC (cm)	79.52 ± 10.52	87.24 ± 10.02	<0.001
BMI (kg/m^2^)	23.14 ± 3.12	25.58 ± 3.59	<0.001
SBP (mmHg)	118.84 ± 13.98	125.69 ± 14.31	0.006
DBP (mmHg)	75.68 ± 9.34	77.56 ± 8.80	0.224
Glucose (mmol/L)	5.26 ± 0.37	5.35 ± 0.49	0.205
Insulin (mU/L)	8.35 ± 3.55	11.16 ± 5.22	0.002
HOMA-IR	1.79 (1.32,2.57)	2.39 (1.56,3.54)	0.003
TG (mmol/L)	0.85 ± 0.22	1.23 ± 0.27	<0.001
TC (mmol/L)	4.08 ± 0.69	4.63 ± 0.90	<0.001
HDL-C (mmol/L)	1.30 ± 0.27	1.22 ± 0.25	0.086
LDL-C (mmol/L)	2.47 ± 0.49	2.95 ± 0.65	<0.001
non-HDL-C (mmol/L)	2.79 ± 0.61	3.41 ± 0.78	<0.001
TRLRs (mmol/L)	0.32 ± 0.16	0.46 ± 0.18	<0.001
ApoAI (mg/dL)	146.0 ± 22.53	143.76 ± 24.72	0.585
ApoB (mg/dL)	65.05 ± 14.10	79.28 ± 17.72	<0.001
ApoCII (mg/dL)	3.98 ± 1.46	4.14 ± 1.39	0.507
ApoCIII (mg/dL)	9.97 ± 3.87	13.02 ± 3.97	<0.001
ApoAI/ApoB	2.29 (1.76,2.71)	1.87 (1.55,2.11)	<0.001
ApoCII/ApoCIII	0.35 (0.27,0.62)	0.32 (0.22,0.43)	0.049

Means ± SD for normally distributed variables or Means (interquartile range) for non-normal distribution.

PNT, postprandial normal triglyceride; PPT, postprandial hypertriglyceridemia; WC, waist circumference; BMI, body mass index; SBP, systolic blood pressure; DBP, diastolic blood pressure; HOMA-IR, homeostasis model assessment of insulin resistance; TG, triglyceride; TC, total cholesterol; HDL-C, high-density lipoprotein cholesterol; LDL-C, low-density lipoprotein cholesterol; non-HDL-C, non-high-density lipoprotein cholesterol; TRLRs, triglyceride-rich lipoprotein remnants; ApoAI, apolipoprotein AI; ApoB, apolipoprotein B; ApoCII, apolipoprotein CII; ApoCIII, apolipoprotein CIII.

### Comparison of Serum Lipid and Apo Levels at Different Time Points

**Figure 1:**
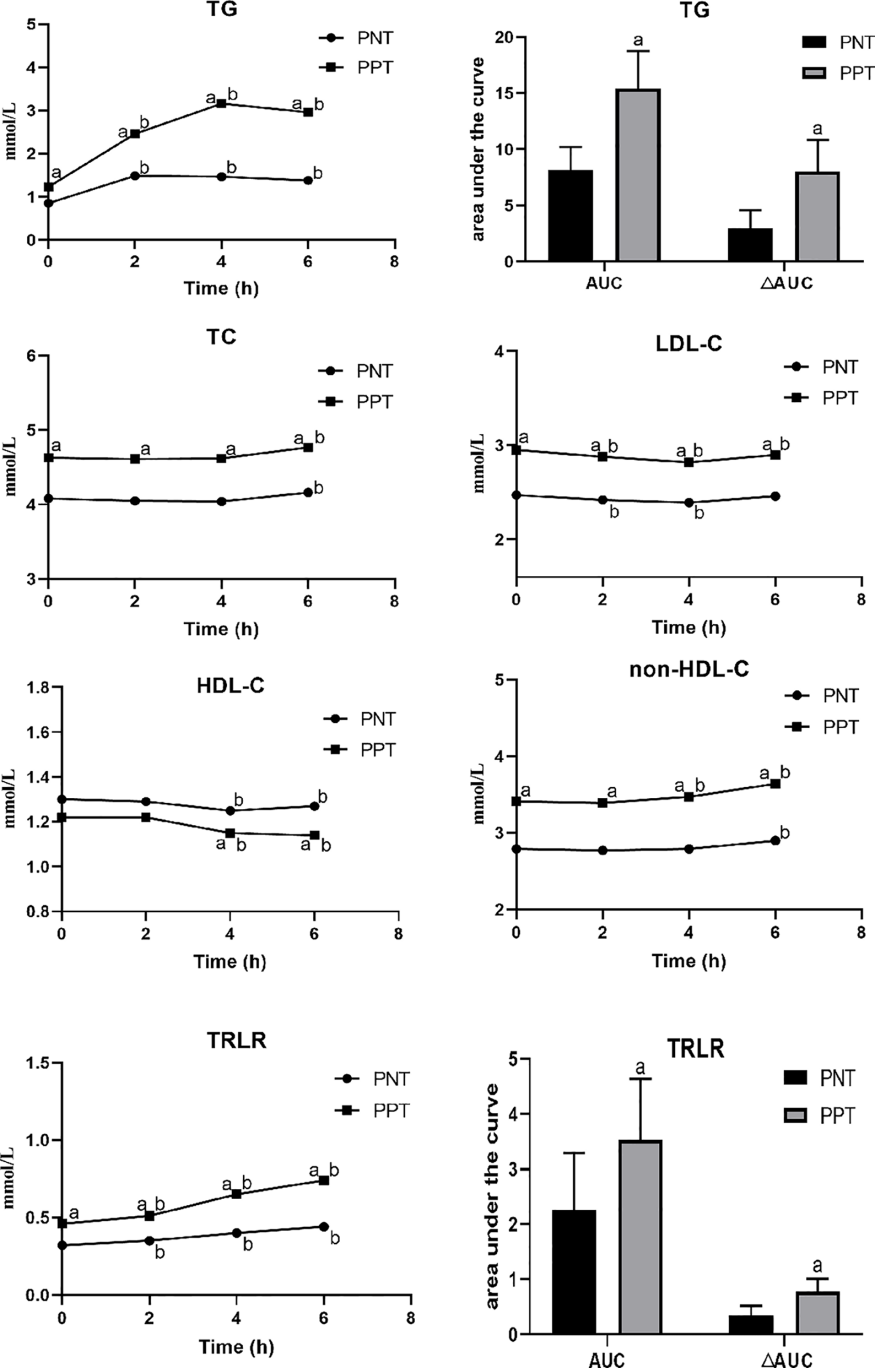
Changes in lipid levels after a high-fat meal in the two groups. PNT, postprandial normal triglyceride; PPT, postprandial hypertriglyceridemia. ^a^ *P *< 0.05, compared with the PNT group; ^b^ *P* < 0.05, compared with the fasting level in the same group.

Postprandial TC, LDL-C, HDL-C, and non-HDL-C levels in both groups at different time points were similar with the fasting levels, while the TG level and TRLRs exhibited obvious increases (**Figure 1**). The levels of TG, TC, LDL-C, non-HDL-C and TRLRs were significantly higher in the PPT group than those in the PNT group at fasting and any time after the high-fat meal (**Table 2**). The postprandial TG level peaked in the PNT group at 2 hours after the meal, while it was significantly higher in the PPT group and peaked at 4 hours after the meal. There was no significant difference in the HDL-C levels of the two groups during fasting and at 2 hours after the meal, while the HDL-C level was lower in the PPT group than that in the PNT group at 4 and 6 hours after the meal. Within 6 hours after the high-fat meal, the TRLRs gradually increased in both groups. The AUC of the TG level and TRLRs in the PPT group, as well as the ΔAUC, were higher than those in the PNT group(*P *< 0.001, **Figure 1**).

**Table 2:** Comparison of serum lipid and apolipoprotein levels at different time points.

	0h	2h	4h	6h
TG (mmol/L)				
PNT (56)	0.85 ± 0.22	1.49 ± 0.48^a^	1.47 ± 0.44^a^	1.38 ± 0.46^a^
PPT (87)	1.23 ± 0.27	2.46 ± 0.68^a^	3.16 ± 0.84^ab^	2.96 ± 0.86^abc^
*P*	<0.001	<0.001	<0.001	<0.001
TC (mmol/L)				
PNT (56)	4.08 ± 0.69	4.05 ± 0.67	4.04 ± 0.70	4.16 ± 0.69^abc^
PPT (87)	4.63 ± 0.90	4.61 ± 0.87	4.62 ± 0.87	4.77 ± 0.92^abc^
*P*	<0.001	<0.001	<0.001	<0.001
HDL-C (mmol/L)				
PNT (56)	1.30 ± 0.27	1.29 ± 0.27	1.25 ± 0.25^ab^	1.27 ± 0.25^ab^
PPT (87)	1.22 ± 0.25	1.22 ± 0.25	1.15 ± 0.23^ab^	1.14 ± 0.23^ab^
*P*	0.086	0.136	0.010	0.002
LDL-C (mmol/L)				
PNT (56)	2.47 ± 0.49	2.42 ± 0.47^a^	2.39 ± 0.48^a^	2.46 ± 0.48^bc^
PPT (87)	2.95 ± 0.65	2.88 ± 0.63^a^	2.82 ± 0.62^ab^	2.90 ± 0.64^ac^
*P*	<0.001	<0.001	<0.001	<0.001
non-HDL-C (mmol/L)				
PNT (56)	2.79 ± 0.61	2.77 ± 0.61	2.79 ± 0.63	2.90 ± 0.62^abc^
PPT (87)	3.41 ± 0.78	3.39 ± 0.75	3.47 ± 0.76^ab^	3.64 ± 0.81^abc^
*P*	<0.001	<0.001	<0.001	<0.001
TRLRs (mmol/L)				
PNT (56)	0.32 ± 0.16	0.35 ± 0.20^a^	0.40 ± 0.19^ab^	0.44 ± 0.19^abc^
PPT (87)	0.46 ± 0.18	0.51 ± 0.18^a^	0.65 ± 0.23^ab^	0.74 ± 0.25^abc^
*P*	<0.001	<0.001	<0.001	<0.001
ApoAI (mg/dL)				
PNT (56)	146.0 ± 22.53	143.75 ± 21.99^a^	142.98 ± 21.94^a^	146.34 ± 22.33^bc^
PPT (87)	143.76 ± 24.72	141.67 ± 22.88^a^	139.25 ± 21.18^ab^	143.91 ± 23.17^bc^
*P*	0.585	0.590	0.313	0.535
ApoB (mg/dL)				
PNT (56)	65.05 ± 14.10	63.73 ± 13.19^a^	63.61 ± 14.03^a^	66.25 ± 13.94^abc^
PPT (87)	79.28 ± 17.72	77.28 ± 17.82^a^	75.33 ± 18.14^ab^	78.44 ± 17.93^bc^
*P*	<0.001	<0.001	<0.001	<0.001
ApoCII (mg/dL)				
PNT (56)	3.98 ± 1.46	8.13 ± 2.95^a^	7.34 ± 2.66^ab^	6.85 ± 2.50^abc^
PPT (87)	4.14 ± 1.39	8.12 ± 2.69^a^	6.77 ± 2.25^ab^	6.41 ± 2.14^abc^
*P*	0.507	0.991	0.175	0.264
ApoCIII (mg/dL)				
PNT (56)	9.97 ± 3.87	20.34 ± 6.72^a^	18.54 ± 6.11^ab^	16.65 ± 5.57^abc^
PPT (87)	13.02 ± 3.97	25.78 ± 7.46^a^	26.65 ± 6.80^ab^	27.41 ± 6.33^abc^
*P*	<0.001	<0.001	<0.001	<0.001
ApoAI/ApoB				
PNT (56)	2.29 (1.76,2.71)	2.28 (1.83,2.69)	2.26 (1.86,2.69)	2.21 (1.81,2.66)^abc^
PPT (87)	1.87 (1.55,2.11)	1.83 (1.56,2.17)^a^	1.85 (1.61,2.22)^ab^	1.88 (1.54,2.15)^bc^
*P*	<0.001	<0.001	<0.001	<0.001
ApoCII/ApoCIII				
PNT (56)	0.35 (0.27,0.62)	0.34 (0.25,0.59)	0.35 (0.27,0.63)	0.38 (0.28,0.64)^abc^
PPT (87)	0.32 (0.22,0.43)	0.31 (0.22,0.41)	0.26 (0.18,0.33)^ab^	0.24 (0.17,0.31)^abc^
*P*	0.049	0.030	<0.001	<0.001

Values are presented as means ± SD.

^a^P<0.05, compared with fasting levels in the same group; ^b^P<0.05, compared with postprandial 2h levels in the same group; ^c^P<0.05, compared with postprandial 4h levels in the same group.

PNT, postprandial normal triglyceride; PPT, postprandial hypertriglyceridemia; TG, triglyceride; TC, total cholesterol; HDL-C, high-density lipoprotein cholesterol; LDL-C, low-density lipoprotein cholesterol; non-HDL-C, non-high-density lipoprotein cholesterol; TRLRs, triglyceride-rich lipoprotein remnants; ApoAI, apolipoprotein AI; ApoB, apolipoprotein B; ApoCⅡ, apolipoprotein CⅡ; ApoCIII, apolipoprotein CIII.

**Figure 2:**
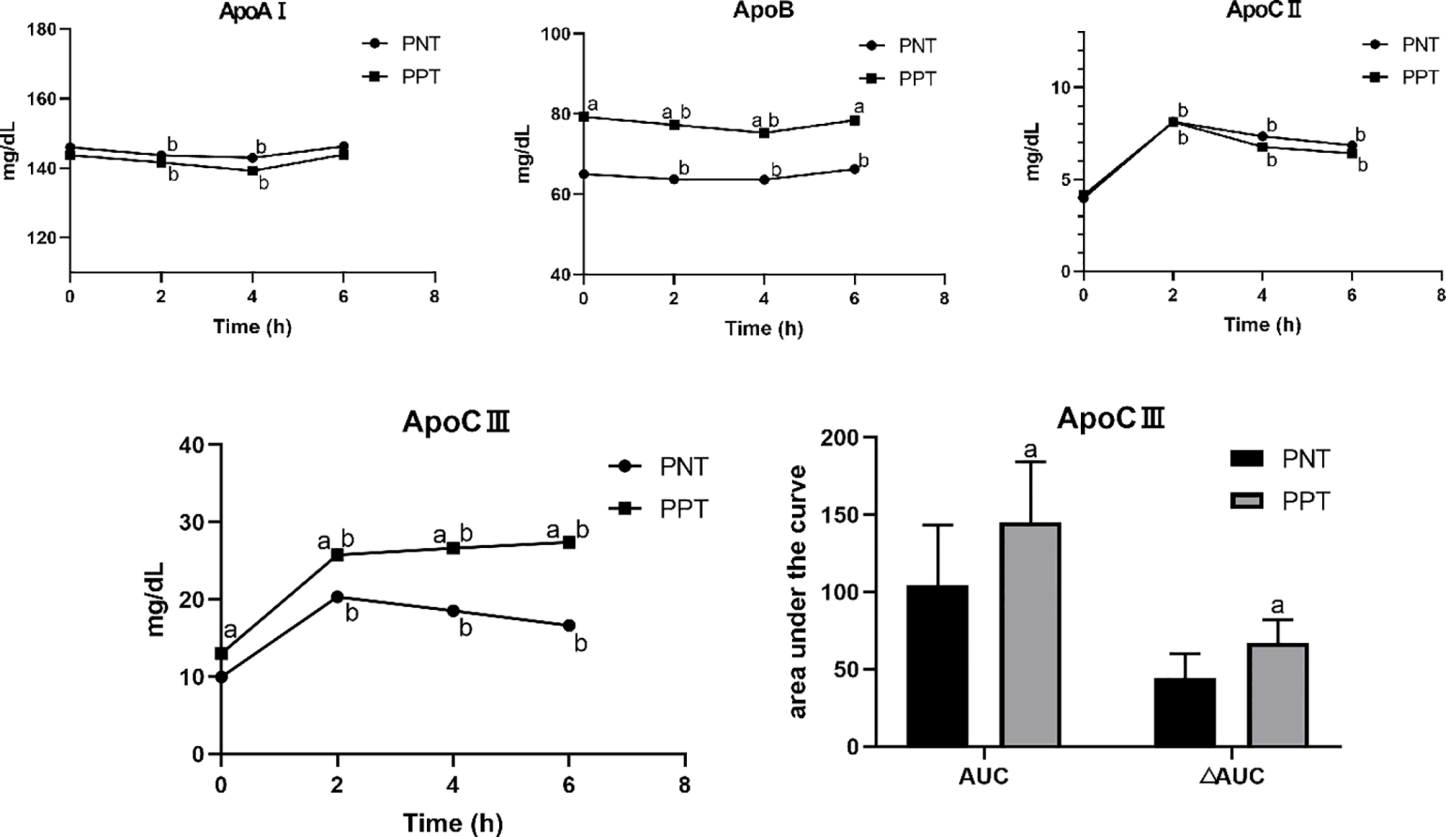
Changes in apolipoprotein levels after a high-fat meal in the two groups PNT, postprandial normal triglyceride; PPT, postprandial hypertriglyceridemia. ^a^ *P *< 0.05, compared with the PNT group; ^b^ *P* < 0.05, compared with the fasting level in the same group.

The postprandial ApoAI and ApoB levels did not significantly change in the two groups, while the ApoCII and ApoCIII levels gradually increased after the meal (**Figure 2**). No significant difference in the ApoAI and ApoCII levels was observed between the two groups at any time point. The ApoCII levels in both groups peaked at 2 hours after the meal and then gradually decreased. The fasting and postprandial ApoB and ApoCIII levels were significantly higher in the PPT group than those in the PNT group. The ApoCIII level in the PNT group peaked at 2 hours after the meal just like the ApoCII, while the ApoCIII level in the PPT group gradually increased within 6 hours after the meal, and the AUC and ΔAUC were higher in the PPT group than those in the PNT group (*P *< 0.001, **Figure 2**). The ApoAI/ApoB and ApoCII/ApoCIII ratios were significantly lower in the PPT group.

### Correlation of Fasting Apo Levels and Other Variables

The fasting ApoAI level was negatively correlated with age, systolic blood pressure, WC, BMI, and HOMA-IR and positively correlated with TC, LDL-C, and HDL-C values (*P *< 0.05) (**Table 3**). The fasting ApoB and ApoCIII levels were positively correlated with age, systolic blood pressure, diastolic blood pressure, BMI, WC, TC, TG, LDL-C, non-HDL-C, and TRLRs values. Additionally, the ApoB and ApoCIII levels were positively correlated with each other (*P *< 0.001, r=0.374). The fasting ApoCII level was not correlated with age, blood pressure, blood lipid or other indicators (*P *> 0.05).

**Table 3:** Correlation of fasting apolipoprotein levels and other variables.

	ApoAⅠ		ApoB		ApoCⅡ		ApoCⅢ	
	r	*P*	r	*P*	r	*P*	r	*P*
Age(years)	-0.199	0.017	0.239	0.004	—	0.474	0.168	0.044
SBP(mmHg)	-0.173	0.039	0.169	0.043	—	0.416	0.206	0.013
DBP(mmHg)	—	0.114	0.242	0.004	—	0.828	0.178	0.033
WC(cm)	-0.204	0.015	0.33	<0.001	—	0.406	0.251	0.002
BMI(kg/m^2^)	-0.264	0.001	0.297	<0.001	—	0.238	0.184	0.027
HOMA-IR	-0.257	0.002	—	0.907	—	0.191	—	0.175
TC(mmol/L)	0.401	<0.001	0.845	<0.001	—	0.568	0.298	<0.001
TG(mmol/L)	—	0.798	0.444	<0.001	—	0.582	0.923	<0.001
LDL-C (mmol/L)	0.183	0.028	0.918	<0.001	—	0.206	0.318	<0.001
HDL-C (mmol/L)	0.887	<0.001	—	0.507	—	0.186	—	0.155
non-HDL-C (mmol/L)	—	0.071	0.921	<0.001	—	0.284	0.371	<0.001
TRLRs (mmol/L)	—	0.924	0.713	<0.001	—	0.869	0.462	<0.001
ApoAI (mg/dL)	—	—	—	0.341	—	0.079	—	0.919
ApoB (mg/dL)	—	0.341	—	—	—	0.501	0.374	<0.001
ApoCII (mg/dL)	—	0.079	—	0.501	—	—	—	0.841
ApoCIII (mg/dL)	—	0.919	0.374	<0.001	—	0.841	—	—

r, correlation coefficient.

SBP, systolic blood pressure; DBP, diastolic blood pressure; WC, waist circumference; BMI, body mass index; HOMA-IR, homeostasis model assessment of insulin resistance; TG, triglyceride; TC, total cholesterol; HDL-C, high-density lipoprotein cholesterol; LDL-C, low-density lipoprotein cholesterol; non-HDL-C, non-high-density lipoprotein cholesterol; TRLRs, triglyceride-rich lipoprotein remnants; ApoAI, apolipoprotein AI; ApoB, apolipoprotein B; ApoCⅡ, apolipoprotein CⅡ; ApoCIII, apolipoprotein CIII.

### Binary Logistic Regression Analysis of Risk Factors of PPT

We used PPT as the dependent variable and fasting indicators as independent variables to perform binary univariate and multivariate logistic regression analyses (**Table 4**). The statistically significant indicators were systolic blood pressure, BMI, WC, TC, TG, LDL-C, non-HDL-C, TRLRs, HOMA-IR, ApoB, ApoCIII, ApoAI/ApoB and ApoCII/ApoCIII. After incorporating the above indicators into the multivariate binary logistic regression analysis model, fasting TG and ApoCIII were independent risk factors for PPT after adjustment (*P *< 0.001, OR=1.350; *P *< 0.001, OR=1.188, respectively).

**Table 4:** Binary logistic regression analysis for assessment of risk factors for postprandial hypertriglyceridemia.

	univariate		multivariate	
	*P*	OR (95% CI)	*P*	OR (95% CI)
Gender	0.994	—	0.687	—
Age (years)	0.323	—	0.621	—
SBP (mmHg)	0.007	1.036(1.010,1.062)	0.587	—
DBP (mmHg)	0.224	—	—	—
BMI (kg/m^2^)	<0.001	1.238 (1.109,1.382)	0.560	—
WC (cm)	<0.001	1.078 (1.038,1.119)	0.692	—
TC (mmol/L)	<0.001	2.325 (1.452,3.722)	0.098	—
TG (mg/dL)	<0.001	1.069(1.045,1.092)	<0.001	1.350 (1.212,1.504)
LDL-C (mmol/L)	<0.001	4.213 (2.131,8.329)	0.755	—
HDL-C (mmol/L)	0.088	—	—	—
HOMA-IR	0.001	1.702 (1.232,2.352)	0.083	—
TRLRs (μmol/L)	<0.001	1.005 (1.003,1.007)	0.956	—
non-HDL-C (mmol/L)	<0.001	3.550 (2.005,6.284)	0.152	—
ApoAI (mg/dL)	0.582	—	—	—
ApoB (mg/dL)	<0.001	1.055 (1.030,1.081)	0.342	—
ApoCII (mg/dL)	0.504	—	—	—
ApoCIII (mg/dL)	<0.001	1.231 (1.111,1.365)	<0.001	1.188 (1.098,1.362)
ApoAI/ApoB	<0.001	0.309 (0.166,0.575)	0.103	—
ApoCII/ApoCIII	0.010	0.161 (0.040,0.649)	0.172	—

OR, odd ratio; CI, confidence interval.

SBP, systolic blood pressure; DBP, diastolic blood pressure; WC, waist circumference; BMI, body mass index; HOMA-IR, homeostasis model assessment of insulin resistance; TG, triglyceride; TC, total cholesterol; HDL-C, high-density lipoprotein cholesterol; LDL-C, low-density lipoprotein cholesterol; non-HDL-C, non-high-density lipoprotein cholesterol; TRLRs, triglyceride-rich lipoprotein remnants; ApoAI, apolipoprotein AI; ApoB, apolipoprotein B; ApoCII, apolipoprotein CII; ApoCIII, apolipoprotein CIII.

## Discussion

In this study, we recruited 143 volunteers to explore the potential predictor of PPT. All the participants took a high-fat meal test and the blood sample was kept at fasting and 2, 4, 6 hours after the meal. After the data analysis, we found that individuals with PPT had higher levels of fasting and postprandial ApoCIII. Further analysis confirmed that ApoCIII is an independent risk factor of PPT. PPT is closely related to atherosclerosis (18, 19) and cardiovascular disease (20), it may also increase the risk of metabolic diseases. A study of first-degree relatives of patients with type 2 diabetes found that although the fasting glucose and blood lipids of these subjects were normal, the postprandial TG levels were significantly higher than in those without a family history of diabetes (21). Our study discovered that ApoCIII is a potential biomarker of PPT and it may be an effective method to treat PPT by controlling ApoCIII levels.

We found that participants in the PPT group not only had a higher postprandial TG level but also had a higher fasting TG level than those in the PNT group. The fasting TG level is an independent risk factor for PPT. Although it may not meet the diagnostic criteria for hypertriglyceridemia, an elevated fasting TG level suggests that lipid metabolism disorders may be present. Therefore, an expert panel recommended that individuals with fasting TG levels of 1–2 mmol/L should take an oral fat tolerance test as an additional investigation for metabolic risk prediction (17). In addition, individuals with PPT also had higher levels of TC, LDL-C, non-HDL-C, and TRLRs. The roles of LDL-C and non-HDL-C have been widely recognized in the occurrence and development of atherosclerosis and cardiovascular disease. Physicians use various lipid-lowering drugs to control LDL-C and non-HDL-C levels to prevent cardiovascular disease (22). Recent studies have also found that a high levels of TRLRs is an important risk factor for cardiovascular disease (23, 24). All of these findings suggest that PPT could increase the risk of cardiovascular disease.

Change of Apos levels can cause dyslipidemia. For example, ApoCII deficiency may increase TG, CM, and VLDL-C levels, while LDL-C and HDL-C levels are reduced (25, 26). But in our study, we didn’t find any significant difference in the ApoCII levels between two groups, so we attributed PPT to other Apos’ change. We found that PPT patients had higher ApoB and ApoCIII levels, not only at fasting but also at postprandial state. The majority of total ApoB is in the form of ApoB100. Even in the postprandial state, the level of ApoB48 particles in healthy individuals is very low (27). Many studies have suggested that ApoB is a more accurate marker of cardiovascular disease than LDL-C or non-HDL-C (28–30). A meta-analysis from 12 independent epidemiological studies including 233,455 participants reported that the relative risk ratios of ischaemic cardiovascular events were highest for apoB (31). Consistent with this finding, ApoB is associated with risk factors for type 2 diabetes, including insulin resistance, glucose-induced hyperinsulinemia and subclinical inflammation (32–34). The increase in the ApoB level in the PPT group indicates that PPT is closely related to the onset of cardiovascular diseases and metabolic diseases. But the postprandial ApoB levels didn’t differ much from fasting levels, even in the PPT patients. The relationship between ApoB and PPT in the univariate logistic regression analysis was eliminated after adjustment for other indicators. So we speculated that ApoCIII may be the main cause of PPT.

ApoCIII is an important regulator in the process of lipoprotein metabolism. Although high concentrations of ApoCIII can inhibit LPL activity *in vitro* (7, 35–37), the amount of ApoCIII present in the VLDL-C of normal or mildly hypertriglyceridemic subjects might not be sufficient to inhibit LPL activity (5). ApoCIII can interfere with the binding of ApoB or ApoE to hepatic receptors (8, 35, 38), thus resulting in delayed catabolism of TRLRs. The TRLRs level significantly increased in the PPT group after the meal, which may be caused by the high ApoCIII level. At high concentrations, ApoCIII inhibits hepatic lipase (9), thus further reducing lipolysis. ApoCIII is mainly synthesized in the liver, and a variety of nutritional components can affect ApoCIII expression. In primary rat hepatocytes and immortalized human hepatocytes, elevated glucose level induced ApoCIII expression in the liver *via* activation of the carbohydrate response element binding protein and hepatic nuclear factor-4α (39). Similarly, elevated postprandial saturated fatty acid levels increased plasma ApoCIII levels and hepatic production in mice and humans (40, 41). In contrast, polyunsaturated fatty acids can inhibit ApoCIII expression in the liver (42), thereby reducing the ApoCIII level. Our study found that fasting ApoCIII is an independent risk factor for PPT. As mentioned above, ApoCIII can affect the metabolism of TG in many pathways, thereby increasing the TG level. The high fasting TG level and the delayed TG metabolism after the high-fat meal may be the result of increased ApoCIII level in PPT patients. This result is also proved by other studies. A research aimed to study the influence of ApoCIII *SstI* polymorphisms in the postprandial response found that homozygosity for the minor allele of the APOC3 *SstI* polymorphism (S2S2) was associated with higher increment of postprandial TG level in metabolic syndrome (43). There is another study showed that inulin can improve PPT of mice by decreasing the mRNA expression levels of ApoCIII in the jejunum (44). ApoCIII also affects hepatic VLDL assembly and secretion, mainly by increasing TG-rich VLDL1 production, while VLDL2 or other ApoB-containing lipoproteins are not affected (45, 46). This may explain the high LDL-C and ApoB levels in the PPT group. Therefore, controlling the ApoCIII level may improve PPT and prevent cardiovascular and metabolic diseases effectively. Many lipid-lowering drugs (such as statins, fibrates, and niacin) can reduce ApoCIII levels, and antisense inhibition therapy for ApoCIII can significantly reduce ApoCIII and TG levels in patients with hyperlipidemia (47).

There are several limitations of this study, including that results of a single high-fat meal test cannot accurately reflect lipid metabolism and may be subject to interference from many factors. In addition, to explore the trend of postprandial Apos levels, ApoB should be further subdivided into ApoB48 and ApoB100. In summary, by comparing the differences in serum ApoAI, ApoB, ApoCII, and ApoCIII levels between individuals with PPT and control participants, we found that ApoCIII is an independent risk factor for PPT. The risk of PPT gradually increases with increasing ApoCIII levels.

## Data Availability Statement

The raw data supporting the conclusions of this article will be made available by the authors, without undue reservation.

## Ethics Statement

The studies involving human participants were reviewed and approved by the ethics committee of Hebei General Hospital, Shijiazhuang, Hebei, China. The patients/participants provided their written informed consent to participate in this study.

## Author Contributions

YG and XH designed and drafted the manuscript. The experimental procedures and data analysis were performed by YG, XH, PT, LR, YT, AS, and LG. GS and JZ gave experimental guidance. All authors contributed to the article and approved the submitted version.

## Funding

This research did not receive any specific grant from funding agencies in the public, commercial, or not-for-profit sectors.

## Conflict of Interest

The authors declare that the research was conducted in the absence of any commercial or financial relationships that could be construed as a potential conflict of interest.
